# Impact of breakfast on daily energy intake - an analysis of absolute versus relative breakfast calories

**DOI:** 10.1186/1475-2891-10-5

**Published:** 2011-01-17

**Authors:** Volker Schusdziarra, Margit Hausmann, Claudia Wittke, Johanna Mittermeier, Marietta Kellner, Aline Naumann, Stefan Wagenpfeil, Johannes Erdmann

**Affiliations:** 1 Else-Kröner-Fresenius Center of Nutritional Medicine, Technical University of Munich, Ismaninger Straße 22, 81675 Munich, Germany; 2 Institute of Medical Statistics and Epidemiology, Technical University of Munich, Ismaninger Straße 22, 81675 Munich, Germany; 3 Graduate School of Information Science in Health Technical University of Munich, Ismaninger Straße 22, 81675 Munich, Germany

## Abstract

**Objective:**

The role of breakfast energy in total daily energy intake is a matter of debate. Acute feeding experiments demonstrated that high breakfast energy leads to greater overall intake supported by cross-sectional data of a free-living population. On the other hand, a large intraindividual analysis has indicated that a high proportion of breakfast to overall intake is associated with lower daily energy intake. To evaluate these apparently contradictory results in greater detail both ways of analysis were applied to the same data set of dietary records.

**Methods:**

On an intraindividual basis total daily energy intake was related to the absolute values of breakfast energy intake or to the ratio of breakfast to overall intake, respectively. Food intake of 280 obese and 100 normal weight subjects was analyzed who recorded over 10 (obese) or 14 (normal weight) consecutive days, respectively.

**Results:**

Increasing breakfast energy was associated with greater overall intake in normal weight and obese subjects. The increasing ratio of breakfast to total daily energy intake was associated with a significant reduction of overall intake on days where post-breakfast energy was significantly reduced. Correlational and multiple regression analysis support the concept that absolute breakfast calories have the strongest influence on daily energy intake.

**Conclusion:**

Reduced breakfast energy intake is associated with lower total daily intake. The influence of the ratio of breakfast to overall energy intake largely depends on the post-breakfast rather than breakfast intake pattern. Therefore, overweight and obese subjects should consider the reduction of breakfast calories as a simple option to improve their daily energy balance.

## Introduction

The role of breakfast consumption in daily energy intake is a matter of debate. In acute feeding experiments Cotton et al. have demonstrated that an energy-rich breakfast does not induce subsequent under-eating to compensate for the extra calories consumed in comparison to a day that started with a breakfast containing 45% less energy [1,2]. Furthermore, a cross-sectional food intake analysis of young adults in Bogalusa has shown that breakfast skipping was associated with substantially lower daily energy intake [3]. On the other hand, the intraindividual analysis of eating habits of free-living subjects has demonstrated that an increasing percentage of breakfast to overall energy intake is associated with lower daily energy intake [4,5].

The results of the studies of Cotton et al. [1,2] and Nicklas et al. [3] are apparently contradictory to the data of de Castro [4,5]. However, a direct comparison of these studies is difficult due to the substantially different methodological approach. The analysis is based either on absolute breakfast calories in relation to total daily intake [1-3] under laboratory or free-living conditions or on the percentage of breakfast to total calories in relation to total daily intake in free living subjects examined intraindividually [4,5]. In this latter group it remains unknown whether the higher ratio of breakfast to total daily energy intake is due to increased breakfast or below average intake of the subsequent meals or a combination of both. This, however, is of interest in the perspective of dietary counselling.

In the present study we have analysed the intraindividual relationship between breakfast and total daily energy intake in a group of 280 obese and 100 normal weight subjects. In both groups data were analyzed on the basis of 1. absolute breakfast energy intake and 2. the ratio of breakfast to overall energy intake, respectively. A detailed analysis of total daily and single meal food intake has been reported recently for both groups [6-10]. The overall energy intake by solid food and beverages was comparable to the age- and gender-related groups of the recent German National Nutrition Survey II [11].

## Research design and methods

A total of 280 overweight and obese patients and 100 normal weight subjects were evaluated. The patients contacted the outpatient clinic of the department of nutritional medicine for the treatment of their weight problem. All had maintained their weight for at least 12 months without any period of intentional or spontaneous weight loss. The normal weight subjects were recruited by advertisement and the selection was made according to age and gender to match the obese group as much as possible. All examinations were performed according to the guidelines of the ethical committee of the Technical University of Munich and in accordance to the principles of the Declaration of Helsinki.

The demographic characteristics are summarized in Table 1. As part of our routine treatment program all patients had to complete a food diary over 10 consecutive days prior to the start of therapy. They were instructed to record in as detailed a manner as possible every item that they either eat or drank, the time they consumed it, the amount they ate and how the food was prepared. To improve the motivation to accurately report their habitual food intake patients were told that an accurate recording of their usual intake improves individually adapted changes of their personal eating habits during subsequent therapy. They were told that the most preferred food items of their usual diet would remain unchanged as much and as long as possible to prevent underreporting especially of snacks with high energy density (ED).

**Table 1:** Demographic characteristics of study population (mean ± SEM)

	Obese	Normal weight
n	280	100
Sex (female/male)	205/75	67/33
Age (years)	45 ± 0.85	42 ± 0.2
Body weight (kg)	108 ± 3.4	67 ± 0.3
BMI (kg/m^2^)	36.6 ± 0.2	22.5 ± 0.1

They were informed that record keeping can produce a change in nutrient intake and they should not try to alter their normal food intake since this could be disadvantageous for the subsequent recommendations during dietary counseling. They should try to record at the time of meal ingestion as much and as often as possible to minimize problems of correctly remembering details and especially the quantity of ingested food items. They should use a scale as often as possible. They were told that the quantity of recorded food items is extremely important for the ensuing therapy. All patients were handed out a booklet with examples of characteristic portion sizes in case of eating out. The same information was given to the normal weight subjects with the exception that they were asked to record intake 14 days.

### Data analysis

Dietary protocols were calculated with the scientific program PRODI 4.5 Expert (Kluthe, Freiburg, Germany). Main meals were divided into breakfast, lunch and dinner. Patients were told to identify main meals as breakfast, lunch and dinner specifically in their records and to indicate the respective time periods. All food items ingested between breakfast and lunch were considered as morning snack (MS). The afternoon snack (AS) comprised the time period between lunch and dinner and the evening snack (ES) considered all food items following dinner. Energy containing beverages were recorded separately. A detailed analysis of the food intake data has recently been reported [6-10].

To assess the intraindividual relationship between breakfast and total daily energy intake each individual's intake was ranked in ascending order on the basis of each day's absolute breakfast energy intake (B kcal). Thus, the lowest value of breakfast energy intake represents the mean value of each individual's lowest intake irrespective of the chronological order of recording. Similarly, data were analyzed on the basis of each individual's ratio of breakfast to total daily energy intake (%B/T kcal), respectively.

For statistical analysis t-test for paired data was employed, where appropriate correction for multiple testing was made according to Bonferroni. Pearson correlation analysis was performed between total daily energy intake and that during breakfast. Multiple regression analysis using stepwise forward and backward variable selection was used to examine the influence of the independent variable breakfast energy, and the respective ratio of this parameter to the corresponding overall intake data on the dependent variable daily energy intake. P-values of 0.05 or less were considered significant. All data were analyzed by using SPSS (version 17.0).

## Results

In ***obese ***subjects the average solid food breakfast energy intake on each individual's lowest day was 121 ± 7.1 kcal and it increased up to 606 ± 19.0 kcal. In part this high degree of variability in energy intake is due to occasional breakfast skipping.

**Figure 1:**
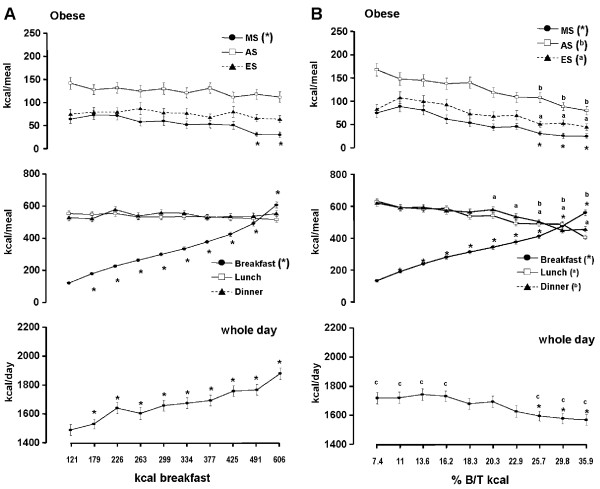
**Relationship between breakfast energy intake (A) or the percentage of breakfast to overall energy intake (% B/T kcal) (B), respectively and whole day, lunch, dinner and snack intake by solid food in 280 obese subjects**. (Mean ± SEM). * = significant difference of p < 0.05 or less vs. the day with the lowest breakfast energy intake or the day with the lowest ratio. ^c ^= significant difference of p < 0.05 or less vs. the whole day intake when analysed on the basis of absolute breakfast calories. MS = morning snack, AS = afternoon snack, ES = evening snack.

The corresponding data of energy intake during the other meals and overall daily intake is shown in Figure 1a. Lunch and dinner intakes were around 550 kcal on all days independent of breakfast energy intake. During the morning snack energy intake decreased significantly on the 2 days with highest breakfast intake while afternoon and evening snack intakes remained unchanged, respectively. Total energy intake rose by ~400 kcal which largely corresponds to the change of breakfast intake.

**Figure 2:**
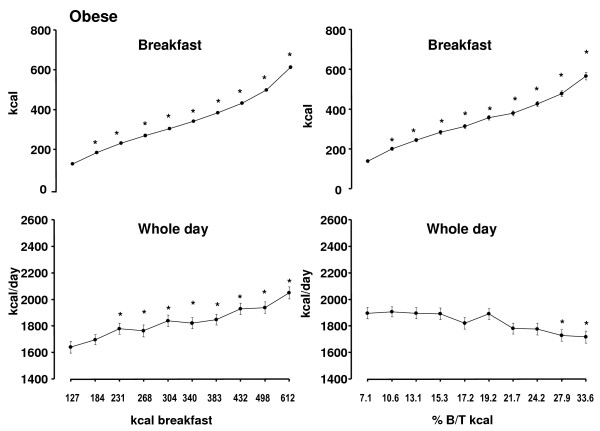
**Relationship between breakfast and whole day, energy intake with inclusion of energy containing beverages (left side) and relationship between the percentage of breakfast to overall intake (% B/T kcal) and whole day and breakfast energy intake with inclusion of energy containing beverages (right side) in 280 **obese subjects **(Mean ± SEM) **. * = significant difference of p < 0.05 or less vs. the day with the lowest breakfast energy intake or the lowest ratio, respectively.

The analysis with consideration of energy containing beverages led to a parallel shift of breakfast and overall energy intake (Figure 2a).

The analysis on the basis of breakfast energy as percentage of total daily energy intake is shown in Figure 1b. With the progressively increasing ratio breakfast calories rose from 134 ± 7.9 to 559 ± 18.2 kcal. Energy intake during all single meals and overall intake decreased steadily. Dinner, snacks and overall intake were significantly lower on the 3 days with the highest ratio, respectively while lunch intake was significantly lower during 5 days (Figure 1b). When beverages were included in the analysis the relationship between breakfast and overall intake remained similar (Figure 2b). The comparison of overall daily intake between the 2 modalities of analysis showed significantly higher energy intake on the 4 days with the lowest ratio compared to the corresponding 4 days with the lowest absolute breakfast intake. Additionally the 3 days with the highest ratio had a significantly lower intake (Figures 1a and 1b).

**Figure 3:**
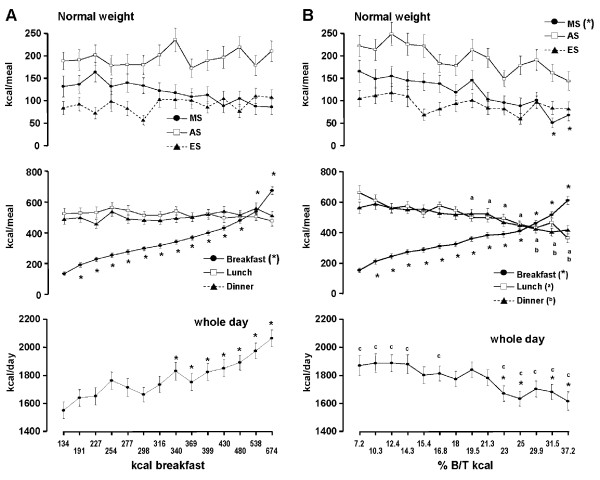
**Relationship between breakfast energy intake **(A) **or the percentage of breakfast to overall energy intake (% B/T kcal) **(B)**, respectively and whole day, lunch, dinner and snack intake by solid food in 100 **normal weight subjects**. (Mean ± SEM) **. * = significant difference of p < 0.05 or less vs. the day with the lowest breakfast energy intake or the day with the lowest ratio. ^c ^= significant difference of p < 0.05 or less vs. the whole day intake when analysed on the basis of absolute breakfast calories. MS = morning snack, AS = afternoon snack, ES = evening snack.

**Figure 4:**
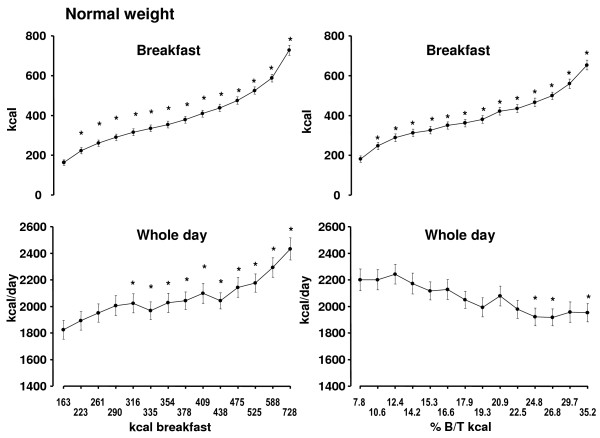
**Relationship between breakfast and whole day energy intake with inclusion of energy containing beverages (left side) and relationship between the percentage of breakfast to overall intake (% B/T kcal) and whole day and breakfast energy intake with inclusion of energy containing beverages (right side) in 100 **normal weight subjects **(Mean ± SEM) **. * = significant difference of p < 0.05 or less vs. the day with the lowest breakfast energy intake or the lowest ratio, respectively.

In the ***normal weight ***group the range of daily breakfast energy intake extended from 134 ± 12.8 to 674 ± 24.6 kcal (Figure 3a). Similar to the obese subjects occasional breakfast skipping is also relevant in the normal weight group. Lunch and dinner energy intake remained constant at 500 to 550 kcal and similarly no significant changes were observed for the three snacks. Total energy intake increased significantly by 500 kcal/d. Similar to the obese group inclusion of beverages leads to a parallel shift of energy intake (Figure 4a).

When the analysis is based on the ratio of breakfast to overall intake breakfast energy was in the range of 152 to 611 kcal (Figure 3b). A significant decrease was observed at lunch, dinner, morning snack and overall daily intake, respectively. During the 5 days with the lowest ratio whole day energy intake was significantly higher compared to the corresponding days of whole intake based on absolute breakfast calories. On the other hand, the 5 days with the highest ratio were significantly lower (Figures 3a and 3b). The addition of beverages to the analysis led to a parallel shift of energy intake without substantially affecting the relationship between breakfast and overall intake (Figuer 4b).

The major food groups responsible for the significant increase of breakfast energy intake between the lowest and highest day (obese/normal weight) were: bread (145/126 kcal), eggs 21/33 kcal), cake (51/132 kcal), yogurt (9/11 kcal), cheese (57/84 kcal), sausages (70/- kcal), marmalade (22/34 kcal), butter (37/42 kcal). Other food groups had per se no significant changes but the additive effect was responsible for the remaining increase of energy intake.

### - correlations and regression analysis

Breakfast energy intake correlated positively and significantly with daily energy intake in both normal weight and obese subjects (Table 2). In the obese group the ratio of breakfast to overall energy intake was also positively correlated with daily energy intake. The correlation coefficients, however, were much smaller than those for the absolute intake data. In the normal weight group the ratio of energy intake was inversely and significantly correlated with daily energy intake.

**Table 2:** Pearson correlation coefficients for the relationship between the various parameters of breakfast intake with daily energy intake in normal weight and obese subjects.

	Obese		Normal weight	
Parameters of breakfast	r	p	r	p
kcal	0.477	<0.0001	0.453	<0.0001
% B/T kcal	0.122	<0.0001	-0.081	0.002

The multiple regression analysis (Table 3) demonstrated in obese subjects a significant positive influence of breakfast energy while the ratio was inversely related to daily energy intake. In the normal weight group breakfast energy and the ratio of breakfast to overall energy intake were significantly related to daily energy intake, respectively. In both groups the strongest influence had absolute breakfast energy intake.

**Table 3:** Variable selection during multiple linear regression analysis with the independent variables breakfast energy (kcal), % B/T kcal and total daily energy intake as the dependent variable in *obese and normal weight subjects*.

	Obese		Normal weight	
	Std. coefficient β	p	Std. coefficient β	p
kcal breakfast	0.861	<0.0001	1.386	<0.0001
% B/T kcal	-0.354	<0.0001	-1.181	<0.0001

## Discussion

The impact of breakfast on total daily energy intake is a matter of debate. In an experimental setting Cotton et al. [1,2] have shown in normal weight subjects that an energy-rich breakfast leads to greater daily energy intake indicating that compensatory undereating during subsequent meals does not occur. This notion is supported by an analysis of food intake in young adults in Bogalusa [3]. It must be kept in mind though that these findings are based on a cross-sectional analysis and accordingly these data represent interindividual differences.

Up to the author's knowledge the present data demonstrate for the first time this relationship in a free-living population of normal weight as well as obese subjects thereby confirming the earlier experimental data of Cotton and colleagues. When analysed intraindividually whole day energy intake increases proportionally to breakfast calories while other meals of the day remain largely unchanged. The only exception is a small reduction of the morning snack on the two days with the highest breakfast energy consumption in the obese group.

In a different approach de Castro [4,5] has demonstrated that a high ratio of breakfast to whole day energy intake is associated with lower overall intakes. The present data also confirm these previous findings. An increased ratio of breakfast to whole day energy intake can be due to an augmented consumption during breakfast or to a reduced intake during all subsequent meals or a mixture of both. The direct comparison of both ways of analysis show that the range of breakfast energy intake is largely similar.

Thus, the significant reduction of daily energy intake on days with a high B/T ratio is mainly the result of below average intakes during the other meals. Moreover, it should be noted that a low B/T ratio is associated with significantly greater overall intake compared to the days with a low absolute intake.

For the purpose of reducing daily energy intake in obesity prevention or treatment, a reduction of breakfast calories can be helpful. High breakfast energy intake is not automatically associated with low whole day intake. On these days substantially reduced energy consumption during post-breakfast meals would be necessary.

With regard to obesity treatment the role of breakfast intake is controversial. Several cross-sectional studies have indicated that breakfast intake is inversely associated with body weight [12-16]. In contrast a large longitudinal study came to the conclusion that a high percentage of breakfast energy is associated with lower weight gain [17]. It must be kept in mind though, that at the beginning of this study a high percentage of breakfast energy was associated not only with the highest overall intake but also with the highest body weight which is in contrast to the results of other cross-sectional studies [12-16]. Unfortunately food intake was assessed only at baseline but not at follow-up. Thus, lower weight gain could be due to a change of eating habits leading to reduced breakfast and overall energy intake over the three years of follow-up.

Moreover, data of the National Weight Control Registry (NWCR) [18] have shown that the majority (78%) regularly eats breakfast which has been suggested to be an important factor for successful maintenance of weight loss. It is important to note, however, that this is not necessarily a causal relationship since the other 22% are also successful weight loss maintainers, despite skipping breakfast regularly or occasionally, respectively. In the present analysis 5 out of 280 obese subjects (1.8%) never had breakfast. 180 patients never skipped breakfast and the other 95 occasionally skipped breakfast. All groups had a similar BMI and total daily energy intake was not different. In the normal weight group total daily energy intake of those with regular breakfast consumption (n = 68) was significantly higher (1826 ± 18.4 kcal/d) compared to the 32 subjects who skipped breakfast occasionally (1691 ± 31.5 kcal/d; p < 0.001) while body weight was not different between these 2 groups, which indicates that energy expenditure has to be considered as well. In this context it is noteworthy that the regular breakfast eaters in the NWCR reported more physical activity.

On the other hand, the only interventional study of the role of breakfast in the treatment of obesity came to mixed results [19]. Baseline breakfast eaters lost more weight when assigned to the no-breakfast treatment group. On the other hand, baseline breakfast skippers lost more weight when assigned to the breakfast-eating treatment group. It should be kept in mind though, that the study population was very small and these very interesting data clearly deserve confirmation in a larger population.

In conclusion the present data demonstrate that higher energy intake at breakfast is highly associated with greater whole day energy intake in normal weight and obese subjects. Therefore low energy intake at breakfast can be helpful to lower daily intake and improve the energy balance during treatment of obesity Whether or not this approach really favours weight loss has to be examined in further interventional studies. At present prevailing data are rather equivocal.

## Competing interests

The authors declare that they have no competing interests.

## Authors' contributions

VS and JE were involved in the study design, data acquisition, data analysis, statistics and manuscript preparation. MH participated in study design; data acquisition and data analysis. CW, JM, MK carried out data acquisition and data analysis. AN and SW performed data analysis and statistics. All authors have read and approved the final manuscript.
